# *Bactrocera dorsalis* in the Indian Ocean: A tale of two invasions

**DOI:** 10.1111/eva.13507

**Published:** 2022-12-01

**Authors:** Pablo Deschepper, Sam Vanbergen, Yue Zhang, Zhihong Li, Issa Mze Hassani, Nausheen Azhaar Patel, Henriette Rasolofoarivao, Sandeep Singh, Suk Ling Wee, Marc De Meyer, Massimiliano Virgilio, Hélène Delatte

**Affiliations:** ^1^ Invertebrates Section Royal Museum for Central Africa Tervuren Belgium; ^2^ College of Plant Protection China Agricultural University Beijing China; ^3^ Key Laboratory of Surveillance and Management for Plant Quarantine Pests Ministry of Agriculture and Rural Affairs Beijing China; ^4^ National Research Institute for Agriculture, Fisheries and Environment, Ex‐CEFADER Mde Comoros; ^5^ Entomology Division Ministry of Agro Industry & Food Security Reduit Mauritius; ^6^ FOFIFA CENRADERU‐DRA Ambatobe Madagascar; ^7^ Department of Fruit Science Punjab Agricultural University Ludhiana India; ^8^ Department of Biological Science and Biotechnology, Faculty of Science and Technology, Centre for Insect Systematics Universiti Kebangsaan Malaysia Bangi Malaysia; ^9^ CIRAD, UMR PVBMT Antananarivo Madagascar

**Keywords:** *Bactrocera dorsalis*, Indian Ocean, invasive species, pest species, phylogeography

## Abstract

An increasing number of invasive fruit fly pests are colonizing new grounds. With this study, we aimed to uncover the invasion pathways of the oriental fruit fly, *Bactrocera dorsalis* into the islands of the Indian Ocean. By using genome‐wide SNP data and a multipronged approach consisting of PCA, ancestry analysis, phylogenetic inference, and kinship networks, we were able to resolve two independent invasion pathways. A western invasion pathway involved the stepping‐stone migration of *B. dorsalis* from the east African coast into the Comoros, along Mayotte and into Madagascar with a decreasing genetic diversity. The Mascarene islands (Reunion and Mauritius), on the contrary, were colonized directly from Asia and formed a distinct cluster. The low nucleotide diversity suggests that only a few genotypes invaded the Mascarenes. The presence of many long runs of homozygosity (ROH) in the introduced populations is indicative of population bottlenecks, with evidence of a more severe bottleneck for populations along the western migration pathway than on the Mascarene islands. More strict phytosanitary regulations are recommended in order to prevent the further spread of *B. dorsalis*.

## 1 INTRODUCTION

In response to globalization, there has been an increased concern surrounding invasive phytophagous insect species, which is motivated by the accumulating economic losses in the fruit trade (Suckling et al., 2013; Welsh et al., 2021). The oriental fruit fly, *Bactrocera dorsalis* (Hendel) (Diptera: Tephritidae), is a major pest species natively found in wet tropical regions of Asia (Clarke et al., 2019; Zeng et al., 2019). The host range of *B. dorsalis* consists of a large variety of plants in both the species' native (Tan & Serit, 1994; Zeng et al., 2019) and invasive range (Hassani et al., 2016; Moquet et al., 2021; Rwomushana et al., 2008). With increasing invasions worldwide, many efforts have been undertaken to eradicate or suppress the species locally (Seewooruthun, Soonnoo, & Alleck, 2000; Vargas et al., 2010; Zeng et al., 2019), including the Sterile Insect Technique (SIT), a method relying on the release of sterile male flies that compete with resident fertile males in order to suppress the target pest population (Dyck et al., 2021). Additionally, *B. dorsalis* is effectively displacing native fruit fly species as has been recently shown by Moquet et al. (2021), drawing the attention of researchers.

Up to now, the genetic population structure of *B. dorsalis* has been studied using microsatellite markers (Kim et al., 2021; Shi et al., 2012) and mtDNA (i.e., COI gene; Ayyasamy & Kamala Jayanthi, 2020; Garzón‐Orduña et al., 2019; Li et al., 2012; San Jose et al., 2018) revealing a rich genetic diversity of *B. dorsalis* in its Asian range. Moreover, results of Garzón‐Orduña et al. (2019) indicate that researchers are only scratching the surface of *B. dorsalis* mitochondrial haplotype diversity. Spatial structuring of *B. dorsalis* populations is generally faint with high levels of admixture over large distances, complicating origin tracing of intercepted flies using molecular techniques (Choudhary et al., 2016; Kim et al., 2021; Shi et al., 2012). However, the weak spatial structure of *B. dorsalis* could also be related to the markers used so far, which may not allow high‐resolution characterization of population structure.

Within the last century *B. dorsalis* has invaded the USA in Hawaii (Vargas et al., 2010), California (Barr et al., 2014), and Florida (Alvarez et al., 2016; Steck et al., 2019; Zeng et al., 2019), was introduced to Africa and Oceania (Vargas et al., 2015), and expanded its range in China (Garzón‐Orduña et al., 2019; Shi et al., 2012). While recent range expansions in India (Choudhary et al., 2016) and several other Asian countries (Zeng et al., 2019) have also been suggested, these statements likely originate from the confounding of “first detection” with “first taxonomic record” where the latter does not imply that the species was previously absent and incomplete knowledge of *B. dorsalis* taxonomic history (Clarke et al., 2019). Furthermore, *B. dorsalis* was first documented in (East) Africa in the Coast Province of Kenya, during routine field surveys by Lux et al. (2003) where it was first inaccurately described as *B. invadens* (Ekesi et al., 2006; Schutze et al., 2015; Zhang et al., 2016). From there, it has rapidly expanded westwards and southwards along the Indian Ocean coast and has reached South Africa in 2010 (Drew et al., 2005; Zeng et al., 2019). During the last decade, *B. dorsalis* managed to be re‐established in Florida in 2015 (Steck et al., 2019) and caused several outbreaks in California during 2006–2012 and 2021 (Barr et al., 2014; USDA APHIS, 2021) after initial eradication. First detections of *B. dorsalis* in Europe occurred in Italy (Nugnes et al., 2018) and France (https://draaf.paca.agriculture.gouv.fr/) in recent years.

Range expansions of *B. dorsalis* are fairly well documented, but little research has been done on the genetic nature of migration pathways of *B. dorsalis* on a regional scale (Garzón‐Orduña et al., 2019; Zeng et al., 2019). Especially information on human‐mediated range expansions, which can be complex in nature (e.g., colonization of oceanic islands) is generally lacking and could provide new information beneficial to sanitary protocols such as improved quarantine systems and better detection and screening methods (Kim et al., 2021; Suckling et al., 2013; Welsh et al., 2021). One example of human‐mediated range expansion is the spread of *B. dorsalis* throughout islands in the Indian Ocean. Here, *B. dorsalis* was first detected in 2005 on Grande Comore, an island part of the Union des Comores (De Meyer et al., 2012). The two other major islands of the country, Anjouan and Mohéli, are assumed to have been colonized later based on monitoring of *B. dorsalis* abundances on the islands (Hassani et al., 2016). Colonization of the Comoros was rapidly followed by an occurrence in the French Overseas Department Mayotte in 2007 (De Meyer et al., 2012), and eventually, *B. dorsalis* reached Madagascar in 2010 (Raoelijaona et al., 2012). The first records of *B. dorsalis* on Mauritius, situated to the east of Madagascar, date back to 1996, where it was declared eradicated after a nationwide eradication program in 1999 (Seewooruthun, Permalloo, et al., 2000; Seewooruthun, Soonnoo, & Alleck, 2000). In 2013, the species was recorded again, but its re‐occurrence was soon followed by a second eradication (Sookar & Deguine, 2016). In 2015, *B. dorsalis* was trapped for the third time in Mauritius, and no large‐scale eradication programs have been pursued since then (Sookar et al., 2020).

Knowledge of the invasion pathway and genetic structure of *B. dorsalis* in the Indian Ocean is lacking and could be beneficial for policymakers (Szyniszewska, 2016). Moreover, the invasion of the islands in the Indian Ocean by *B. dorsalis* could be a prime example of founder effects as a byproduct of island hopping (Mayr, 1942; Sendell‐Price et al., 2021; Tinghitella et al., 2011). Colonized populations may undergo a step‐wise reduction in genetic diversity relative to their sources and rapid differentiation from source populations (Sendell‐Price et al., 2021; Tinghitella et al., 2011).

Given the different dates for historical recordings on several islands in the western Indian Ocean and possible discrepancies between the detection timeline and actual range expansion, we aim to explore the colonization pathway of this invasive species and its genetic relationship with the population in its native Asian range and invaded range on mainland Africa. Furthermore, we expect to pick up an erosion of genetic diversity within the invasive range, especially on oceanic islands, and a buildup of homozygous genomic regions within the invasive range compared with the genetic source. In order to test the above, we performed whole genome (re‐)sequencing of *B. dorsalis* samples collected in the Indian Ocean, adjacent continental Africa, and Asia. A multi‐pronged analytic approach was employed, including an allele frequency‐based tree building algorithm and network theory on SNP markers.

## 2 MATERIALS AND METHODS

### 2.1 Sampling, sequence filtering, and genotyping

For this study, we used 237 *B. dorsalis* specimens obtained from 21 populations in Asia (seven populations, 66 specimens), Africa (three populations, 30 specimens), and several islands in the Indian Ocean (11 populations and 141 specimens): Grande Comoro, Anjouan, Mohéli (Comoros W, E, and C, respectively), Mayotte, Madagascar, and the Mascarene islands (Mauritius and Réunion) using methyl eugenol traps. More than one population was included for Mayotte, Madagascar, and Réunion (two, three, and two, respectively; Table S1). Additionally, seven specimens of one *Bactrocera zonata* population collected in Tel Aviv, Israel, were included to serve as an outgroup for phylogeographic tree reconstruction. Samples were collected between 2016 and 2021, with the exception of those from Burundi and Malawi, which were collected in 2011. See Table S1 for further details on coordinates, number of samples, and collection year per location.

After morphological species identification (https://fruitflykeys.africamuseum.be/), DNA extraction was performed using the Qiagen Blood & Tissue extraction kit following the manufacturer's recommendations. The Asian and African samples were extracted with a different kit: the Promega Wizard SV Genomic DNA Purification System. Elution was carried out with 120 μl buffer AE. DNA concentration was evaluated using a Qubit fluorometer (Thermo Fisher Scientific). Samples that did not meet the minimum concentration guideline of 10 ng/μl were reconcentrated using a Concentrator Plus (Eppendorf). DNA samples were then submitted for whole genome sequencing (150 bp paired‐end) on the NovaSeq 6000 platform with an average input size of 350 bp and a minimum output of 6 Gb (rendering a 12.7× theoretical coverage of the *B. dorsalis* reference genome).

Assessment of the general quality of the raw reads was performed using “fastQC” (https://github.com/s‐andrews/FastQC). Reads were then quality trimmed with “fastp” (Chen et al., 2018), discarding read pairs of which more than 2% of the bases failed to meet a quality threshold of 10. Trimmed reads were aligned to the *B. dorsalis* reference genome (GenBank assembly accession: GCA_020283865.1) using the bwa‐mem command from the burrows‐wheeler aligner tool (Li & Durbin, 2009). Variant calling was performed using the GATK *Haplotypecaller* algorithm implemented in “Elprep” (Herzeel et al., 2021). “ElPrep” is a tool designed as an in‐memory and multithreaded toolset to fully take advantage of the processing power available with modern servers and consists of a 5‐step variant calling best practices pipeline. After individual variant calling, gVCF files were combined using the GATK *CombineVCFs* command (McKenna et al., 2010). Joint variant calling was performed separately for each of the six chromosomes using GATK *GenotypeGVCFs*, and chromosome VCF files were merged afterward. Joint calling allows for the rescue of low‐coverage genotypes in one sample when another sample provides evidence supporting the existence of the genotype. Biallelic variants were hard filtered using GATK *VariantFiltration* using the recommended settings (quality‐by‐depth ratio (QD) < 2.0 || read mapping quality (MQ) < 40.0 || probability of strand bias (FS) > 60.0 || symmetric odds ratio (SOR) > 3.0 || MQRankSum < −12.5 || ReadPosRankSum < −8.0). Filtering on QD normalizes QUAL by the number of reads supporting every SNP and is preferred above filtering quality and depth in individual steps. Samples with missingness larger than 20% were omitted from further analysis. Furthermore, SNPs with more than two alleles, a minimum depth and minor allele count lower than three and a missingness higher than 10% were omitted from further analysis using “VCFtools” (Danecek et al., 2011). Finally, to account for the effects of linkage between SNPs in the datasets, we pruned the VCF file based on a pairwise correlation threshold of 0.1 and a window size of 10,000 bases using the package “SNPRelate” (Zheng et al., 2012) implemented in R (version 4.0.3).

### 2.2 Population genetic parameters

Multiple estimates of genetic diversity were calculated using the same filters as stated above, but no minor allele filtering or linkage pruning was applied in order to best approximate the real genomic diversity. We used “STACKS populations” (Catchen et al., 2013) to estimate observed heterozygosity (*H*_O_), expected heterozygosity (*H*_E_), nucleotide diversity (*π*), and the number of private alleles (*A*_PR_) at the population level. A measure of genetic differentiation (*F*_ST_) between population pairs was calculated using the same software. Lastly, we attributed populations to a higher level grouping (i.e., Asia, Africa, the Comoros islands, Mayotte, Madagascar, and the Mascarene islands) based on admixture results and geographical borders and repeated the estimation for all genetic parameters at this level.

### 2.3 Runs of homozygosity

To infer the demographic history and more specifically, historical bottlenecks, we estimated the total number (NROH) and total length (SROH) of long runs of homozygosity (ROH) using “bcftools” (Danecek et al., 2021). Different demographic scenarios cause different ROH signatures (Ceballos et al., 2018), hence, ROH analyses are increasingly being used in population genomic studies (e.g., in the range‐restricted Pyrenean Desman, Escoda & Castresana, 2021; Swedish wels catfish, Jensen et al., 2021 and in killer whales, Foote et al., 2021). Since ROH are sensitive to minor allele frequency (MAF) filtering and linkage pruning, such filters were not applied before calculating the total number of ROH and the total summed length of ROH (Meyermans et al., 2020). To test for significant differences between the six geographical groups, a parametric linear model was carried out using SROH as the predictor variable. A *p‐*value was calculated for every pairwise comparison between geographical groups, and a Tukey's range test was applied to correct for multiple testing using the package “mixlm” (https://github.com/khliland/mixlm) implemented in R.

### 2.4 Admixture analysis and PCA

To infer admixture between samples across the whole dataset, ancestry coefficients for *K* number of ancestral populations were calculated using the *sNMF* function (Frichot et al., 2014) of the “LEA” package (Frichot & François, 2015) implemented in R. *K* varied from 2 to 10 while performing 10 replicate runs for each *K*. For each value of *K*, the best run was selected based on the cross‐entropy criterion. Ancestral proportions (*Q* values) were then visualized for *K* = 2 up to the value of *K* with the lowest cross‐entropy level + 1 to ensure that further subdivision is not meaningful using the R package “ggplot2” (Wickham, 2017).

In order to investigate and visualize the partitioning of genetic variation, an analysis of principal components (PCA) was performed using the *snpgdsPCA* function of the R package “SNPRelate” (Zheng et al., 2012). To test for significant differences between geographical groups, a permutational ANOVA (permanova) was carried out using the first seven eigenvectors as predictor variables and employing 10,000 permutations using the *pairwise.adonis2* function of the “pairwiseAdonis” package (https://github.com/pmartinezarbizu/pairwiseAdonis). A *p‐*value was calculated for every pairwise comparison, and the Benjamini–Hochberg method (Benjamini & Hochberg, 1995) was applied to correct for multiple testing.

### 2.5 PoMo phylogenetic tree

In order to infer a population‐level phylogenetic tree, a Polymorphisms‐aware phylogenetic Model (PoMo) was constructed (De Maio et al., 2015). This approach is well‐suited for tree estimation in scenarios of incomplete lineage sorting and accounts for ancestral variation while being computationally more efficient than other methods.

First, a VCF file was constructed, including all *B. dorsalis* individuals, and the *B. zonata* individuals as the outgroup. The outgroup individuals were variant called in a separate cohort and filtered in an identical manner. Missing data filters were not applied for the outgroup.

Secondly, *bcftools consensus* (Li, 2011) was used to create a whole genome alignment in fasta format using the *B. dorsalis* reference genome (GenBank assembly accession: GCA_020283865.1). The alignment was then converted to a “counts file” containing the allele counts per population for each site with the publicly available python library “cflib” (https://github.com/pomo‐dev/cflib). Sites with missing data were subsequently omitted from the count's file. To account for the assumption of independent sites, 1 million sites across the whole genome were randomly selected of which 8680 were variant sites. Based on these data, we employed a generalized time‐reversible polymorphism‐aware evolutionary model with eight rate heterogeneity categories (GTR + G8 + P; Schrempf et al., 2019) to reconstruct a maximum‐likelihood phylogeny in “IQ‐tree” (Nguyen et al., 2015). This substitution model features an expanded nucleotide state alphabet so that taxa can have polymorphic states, and thus extends traditional models that are based on nucleotide states that are fixed within a taxon (Schrempf et al., 2016). One‐thousand ultrafast bootstrap trees were constructed to calculate node support (Hoang et al., 2018). The resulting tree reconstruction was visualized with ITOL (Letunic & Bork, 2021).

### 2.6 Kinship networks

As a flexible and highly scalable approach for analyzing individual‐based genetic relationships, a kinship network was constructed. The advantage of using an individual‐based metric over a population‐based one such as *F*_ST_ is that no a priori assumptions are made about (meta‐)population structure and thus allowing the data to directly inform the fine‐grain genetic structure (Jones & Manseau, 2022). Kinship is ideally suited to infer the impact of recent events on relationships between individuals (such as captive breeding programs; Escoda et al., 2019) but is also applicable to wild populations without prior knowledge of demography, shared ancestry, or pedigree (Staples et al., 2014).

Kinship was estimated using the *snpgdsIBDMLE* function within the R package “SNPRelate” (Zheng et al., 2012) applying the Expectation–Maximization (EM) algorithm for exploring the maximum value of the log‐likelihood function (Milligan, 2003; Zheng et al., 2012). Two networks were created. The first network includes all edges with edge weight in proportion to the respective kinship value. A second network was constructed by extracting the backbone structure of the network, simplifying the network by retaining the most important edges, using the disparity filter algorithm by Serrano et al. (2009) implemented in the R package “backbone” (available at: https://CRAN.R‐project.org/package=backbone). By imposing a significance level *α* = 0.05 or 0.10, the links that carry weights that are not different than under the null hypothesis can be pruned from the network. This method is preferred above a global weight threshold pruning method and reveals the multilayered and often complex nature of the network (Jones & Manseau, 2022). Pruning at a small value of *α* reveals those edges that constitute a central part of the network backbone while pruning at a higher value of *α* can reveal the more shallow substructures within the network. Nodes without edges are not shown. Node degree is calculated as the number of edges surrounding the respective node.

## 3 RESULTS

On average, 56,655,392 reads were produced per sample with an average number of mapped reads equal to 48,252,941 (85.17% mapping success). After SNP calling and application of GATK hard filtering, 140,132,105 SNPs remained. Five samples were omitted from further analysis based on missingness larger than 20% (three samples from Reunion, one sample from Burundi, and one sample from Sri Lanka). Consecutive filtering at minimum depth and minor allele count higher than or equal to three, site missingness lower than 10% and linkage pruning rendered 909,776 biallelic SNPs for further PCA and kinship network analysis.

### 3.1 Population genetic parameters

Samples from Asia exhibited the highest nucleotide diversity (*π* = 0.0183) and the highest number of private alleles (1,633,403) compared with all other regions (Table 1). The number of private alleles for every Asian population separately was high and relatively evenly distributed (average = 215,444, SD = 41,516). Within the Asian group, Telangana (India E, Table S1) showed the highest nucleotide diversity (*π* = 0.0192, Table 2). The African region and the Comoros had the second highest nucleotide diversity (*π* = 0.0157 for both) followed by Madagascar (*π* = 0.0148), Mayotte (*π* = 0.0147), and the Mascarenes (*π* = 0.0123; Table 1). Within the African group, Kenya showed the highest diversity (*π* = 0.0162), whereas Malawi (*π* = 0.0151) and Burundi (*π* = 0.0149) were ranked below the Comoros. Group‐wise heterozygosity (*H*_O_) varied between 0.0165 (Asia) and 0.0113 (Mascarenes). Standard errors were equal to or smaller than 0.00005 for all parameters.

**TABLE 1:** Genetic parameters for the group level.

Group	#Individuals	*H*_E_	*H*_O_	*π*	*A*_PR_
Asia	65	0.0182	0.0165	0.0183	1,633,403
Africa	29	0.0154	0.0136	0.0157	32,875
Comoros	45	0.0155	0.0136	0.0157	44,288
Mayotte	20	0.0143	0.0145	0.0147	13,108
Madagascar	38	0.0146	0.0134	0.0148	37,854
Mascarenes	35	0.0122	0.0113	0.0123	164,730

**TABLE 2:** Genetic parameters on the level of populations.

Population	#Individuals	*H*_E_	*H*_O_	*π*	*A*_PR_
China	10	0.0174	0.0167	0.0183	249,212
India E	6	0.0176	0.0173	0.0192	155,451
India S	10	0.0159	0.0167	0.0168	175,864
India N	10	0.0173	0.0161	0.0182	240,583
Sri Lanka	9	0.0160	0.0154	0.0169	173,927
Malaysia	10	0.0172	0.0166	0.0181	252,907
Thailand	10	0.0177	0.0170	0.0186	260,164
Kenya	10	0.0154	0.0157	0.0162	11,117
Malawi	10	0.0143	0.0130	0.0151	8538
Burundi	9	0.0140	0.0117	0.0149	7021
Comoros E	15	0.0154	0.0144	0.0160	16,892
Comoros W	15	0.0147	0.0131	0.0152	12,184
Comoros C	15	0.0146	0.0132	0.0151	10,913
Mayotte W	10	0.0135	0.0148	0.0142	6270
Mayotte E	10	0.0137	0.0143	0.0144	6548
Madagascar E	15	0.0140	0.0135	0.0145	13,155
Madagascar N	15	0.0142	0.0132	0.0147	14,239
Madagascar S	8	0.0129	0.0132	0.0138	7972
Réunion W	15	0.0120	0.0116	0.0124	42,483
Réunion E	5	0.0109	0.0114	0.0122	15,497
Mauritius	15	0.0115	0.0111	0.0119	35,972

Genetic differentiation (*F*_ST_) was 0.0089, 0.0090, 0.0092, 0.0093, and 0.0070 between Asia and Africa, Comoros, Mayotte, Madagascar, and the Mascarenes, respectively (Table 3). Genetic differentiation was high between pairwise comparisons of the Mascarenes and Mayotte (0.0416), Africa (0.0345), Comoros (0.0311), and Madagascar (0.0349) (Table 3). Pairwise differentiation at the population level can be found in Table S2.

**TABLE 3:** Pairwise *F*_ST_ values between regional groups.

Region	Asia	Africa	Comoros	Mayotte	Madagascar	Mascarenes
Asia		0.0089	0.0090	0.0091	0.0093	0.0070
Africa			0.0111	0.0216	0.0187	0.0345
Comoros				0.0145	0.0141	0.0311
Mayotte					0.0141	0.0416
Madagascar						0.0349

### 3.2 Runs of homozygosity

**FIGURE 1:**
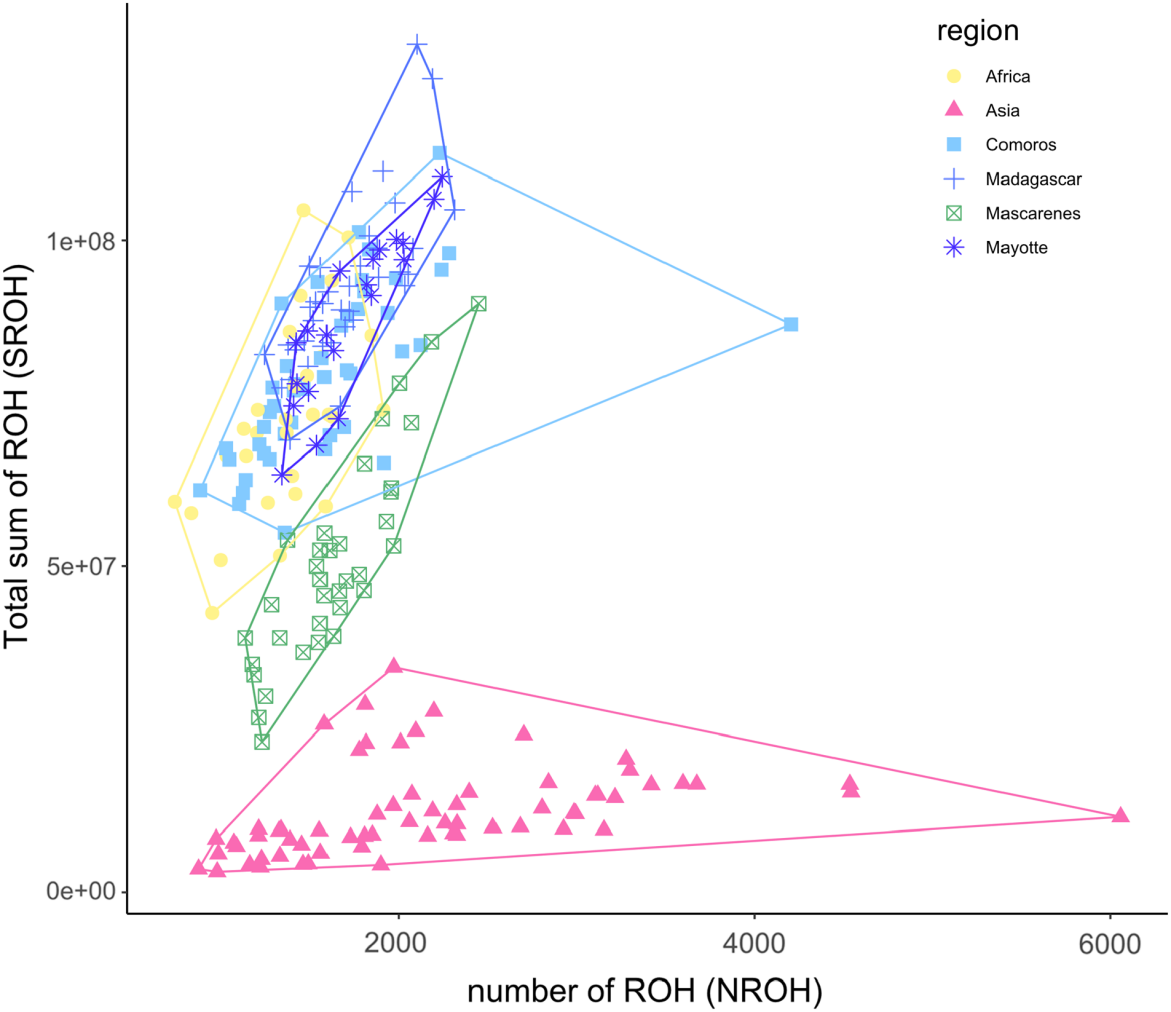
Number of runs of homozygosity (NROH) and total sum of lengths of runs of homozygosity (SROH) for *Bactrocera dorsalis* in six main geographical regions. Higher NROH coupled with a higher SROH is indicative of stronger bottlenecks while a higher SROH indicates more profound inbreeding (Ceballos et al., 2018). A linear regression line is plotted.

Asia was the region with the highest number of ROH (2200 ± 122) while the number of ROH (NROH) was similar and lower for the rest of the regions (Africa: 1366 ± 52.8, Comoros: 1614 ± 78.2, Mayotte: 1729 ± 60.9, Madagascar: 1699 ± 42.7, Mascarenes: 1639 ± 53.1, Figure 1). The summed length of ROH (SROH) was significantly lower for Asia (12,451,121 ± 847,024) when compared to the rest of the regions (average across regions: 76,443,349 ± 2,423,295, Tukey contrasts test: *p* < 0.001), suggesting less inbreeding (Ceballos et al., 2018, Figure 1). Significant differences for SROH were found for all comparisons between regions (*p* < 0.001) except between Comoros–Mayotte (*p* = 0.067), Africa–Comoros (*p* = 0.14), and Madagascar–Mayotte (*p* = 0.79). See Table S3 for the full results of the post hoc comparisons.

### 3.3 Admixture analysis, PCA, and PoMo tree

**FIGURE 2:**
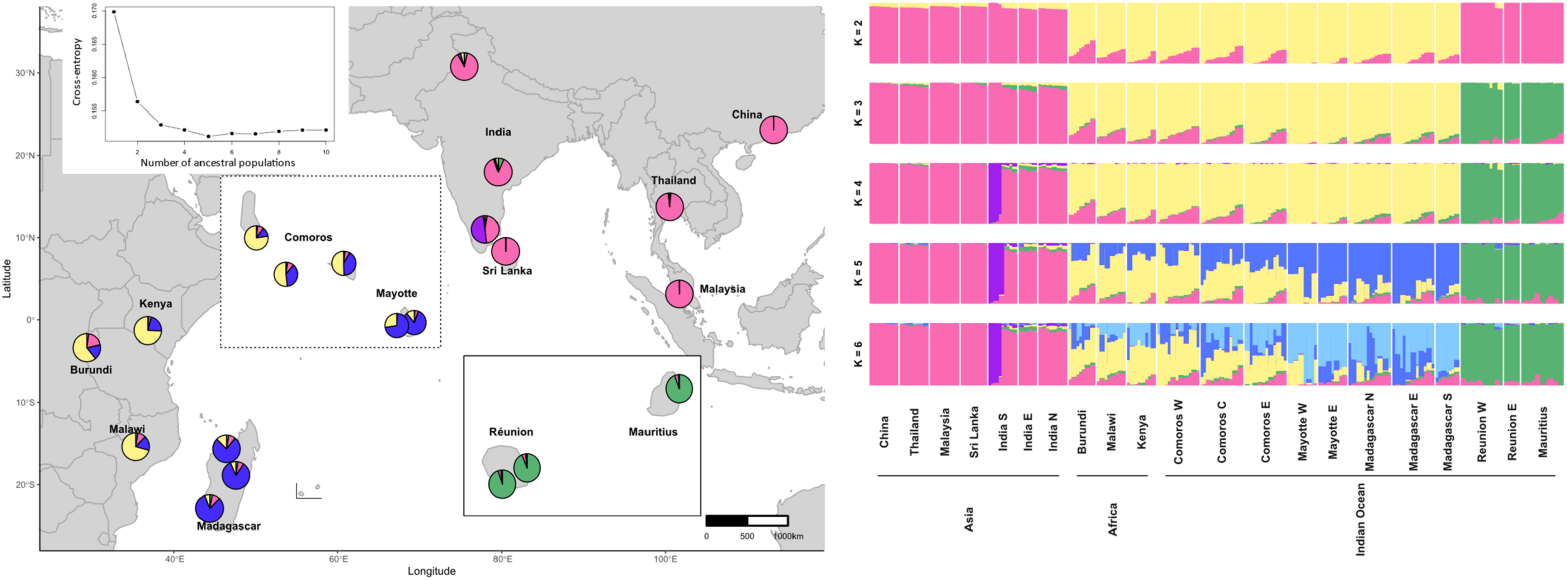
Ancestry coefficients resulting from the sNMF analysis for *Bactrocera dorsalis* in the study region showing the presence of four distinct clusters as represented on a geographical map for *K* = 5 (a) and in a barplot (b). Cross‐entropy for every value of *K* is displayed in the top left corner. A first cluster contains all Asian *B. dorsalis* apart from five samples collected in Tamil Nadu (India S, Table S1), which form a cluster on their own. A third cluster exhibits a clinical pattern and contains individuals from Africa, all three of the Comoros islands, Mayotte, and Madagascar while a fourth cluster encompasses all individuals from the Mascarenes (Mauritius and Reunion).

**FIGURE 3:**
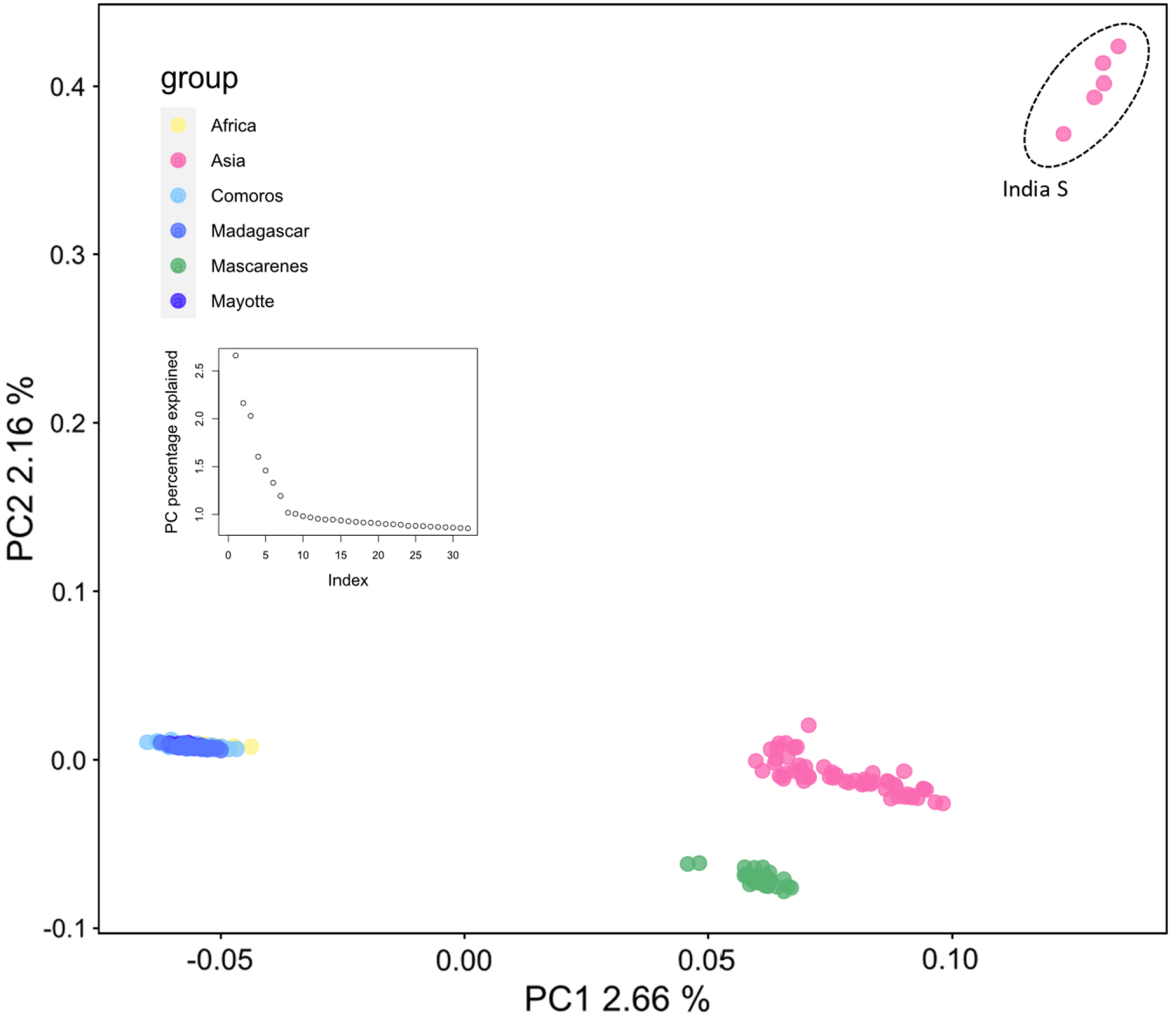
Analysis of principal components (PCA). Results of PCA are indicating that genetic variation can be partitioned into four well‐defined clusters. A first cluster contained all Asian *Bactrocera dorsalis* exclusively. A second cluster contained individuals from Africa, all three of the Comoros islands, Mayotte, and Madagascar, and a third cluster encompassed all individuals from the Mascarenes (Mauritius and Reunion). An additional fourth cluster comprised five out of the 10 individuals sampled in Tamil Nadu, India (India S, Table S1).

The sNMF‐based estimates of shared ancestry point toward the presence of four distinct genetic clusters with *K* = 5 as the optimal *K* according to the level of cross‐entropy (Figure 2). The first cluster contains all Asian samples, excluding five samples from Tamil Nadu (India S, Table S1), which form a cluster on their own. A third cluster suggests a clinal pattern from the African mainland toward Madagascar and contains individuals from Africa, all three Comoros islands, Mayotte and Madagascar. A fourth cluster encompasses all individuals from Mauritius and Réunion (Figure 2). Results from the PCA corroborate the presence of four genetic clusters with each of the clusters coinciding with results from the admixture analysis considering *K* = 4 or larger (Figure 3). Interestingly, five out of the 10 samples from Tamil Nadu (India S, Table S1) are forming a distinct cluster, agreeing with results from the admixture analysis (Figure 2). Individuals from Africa were more closely related to individuals sampled in the Comoros, corroborating the clinal pattern seen in the admixture type analysis. The first and second principal components explained 3.58% and 2.57% of total variations, respectively. The permanova showed significant structuring (*p*_adj_ < 0.05) for all but one pairwise comparison between the six major geographical groups. There was no observed significant difference between Mayotte and the Comoros (*p* = 0.105).

**FIGURE 4:**
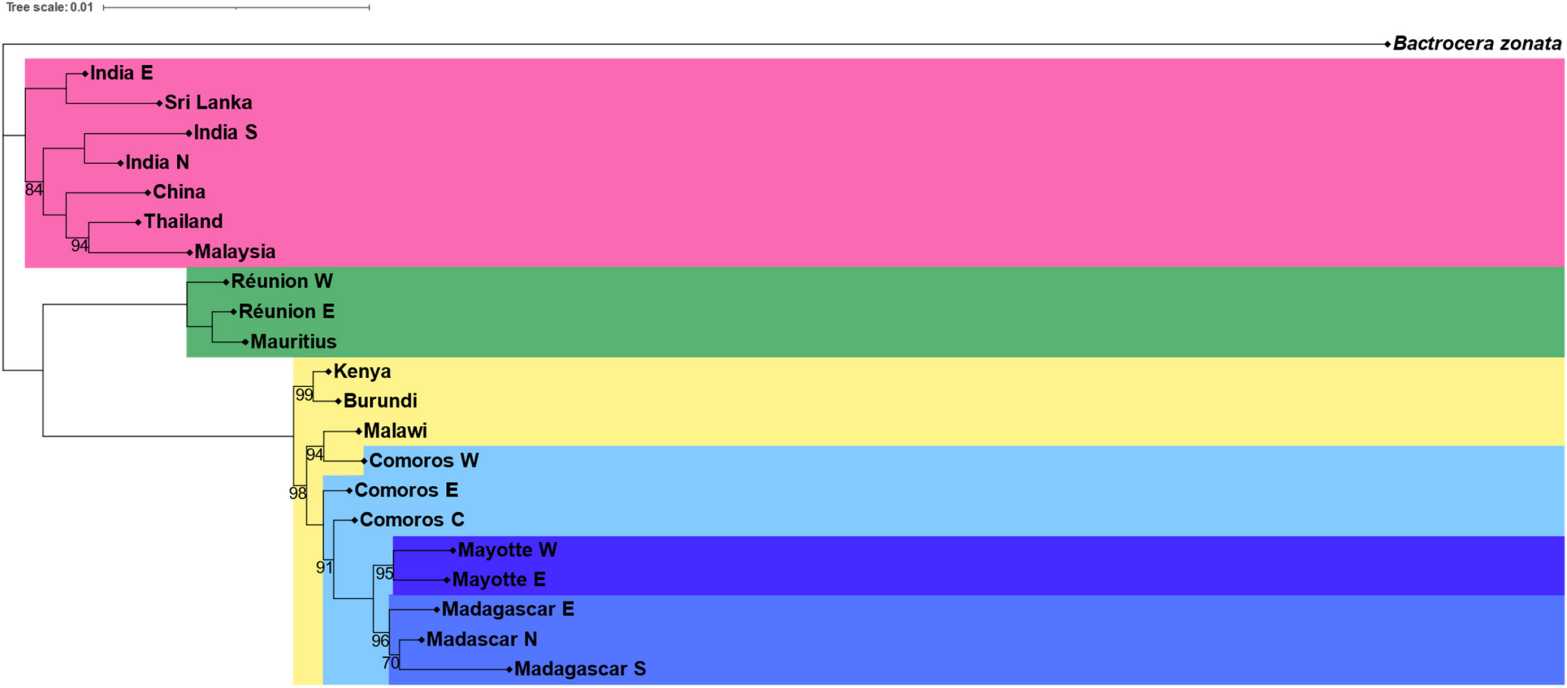
PoMo phylogenetic tree for *Bactrocera dorsalis* in the Indian Ocean with the inclusion of Asian and African populations and *Bactrocera zonata* as an outgroup. Different colors represent the six geographical regions (from top to bottom: Asia, the Mascarenes, Africa, the Comoros, Mayotte, and Madagascar). Only bootstrap values below 100 are shown.

Overall, the topology of the PoMo tree was strongly supported based on bootstrap values (Figure 4). The phylogeography of *B. dorsalis* in the Indian Ocean encompassed two major clades. One clade contained samples from Mauritius‐Réunion (eastern clade) and was paraphyletic to a clade containing the African mainland populations together with the rest of the island populations (western clade). The deep divergence between the western and the eastern clade suggested different origins. Within the western clade, a hierarchical structuring can be observed that coincides with an eastwards direction of invasion from the African mainland (Kenya, Burundi) toward Mayotte and Madagascar. Comoros W was the most basal island within the Comoros and is the most related to the African mainland populations. The rest of the Comoros islands together with Mayotte and Madagascar are paraphyletic to Comoros W (Figure 4).

### 3.4 Kinship networks

Estimated kinship between all dyads was 0.013 on average with a standard deviation of 0.022. A first (kinship >0.25) and second (kinship >0.125) degree of relationship occurred eight and 80 times, respectively, in a total of 26,796 combinations. First‐degree relationships occurred four times in India S, two times in Mauritius, once in Mayotte E and once in Mayotte W. Second‐degree relationships were also exclusively detected between individuals belonging to the same population and could primarily be found in the Western group (28 in Madagascar, 20 in Mayotte and two in the Comoros), followed by the Mascarenes (11 in Mauritius and nine in Réunion) and Asia (10 in India S). The average degree (number of edges per node) within the pruned network (*p* ≤ 0.05) was 4.93 with a standard deviation of 3.40.

**FIGURE 5:**
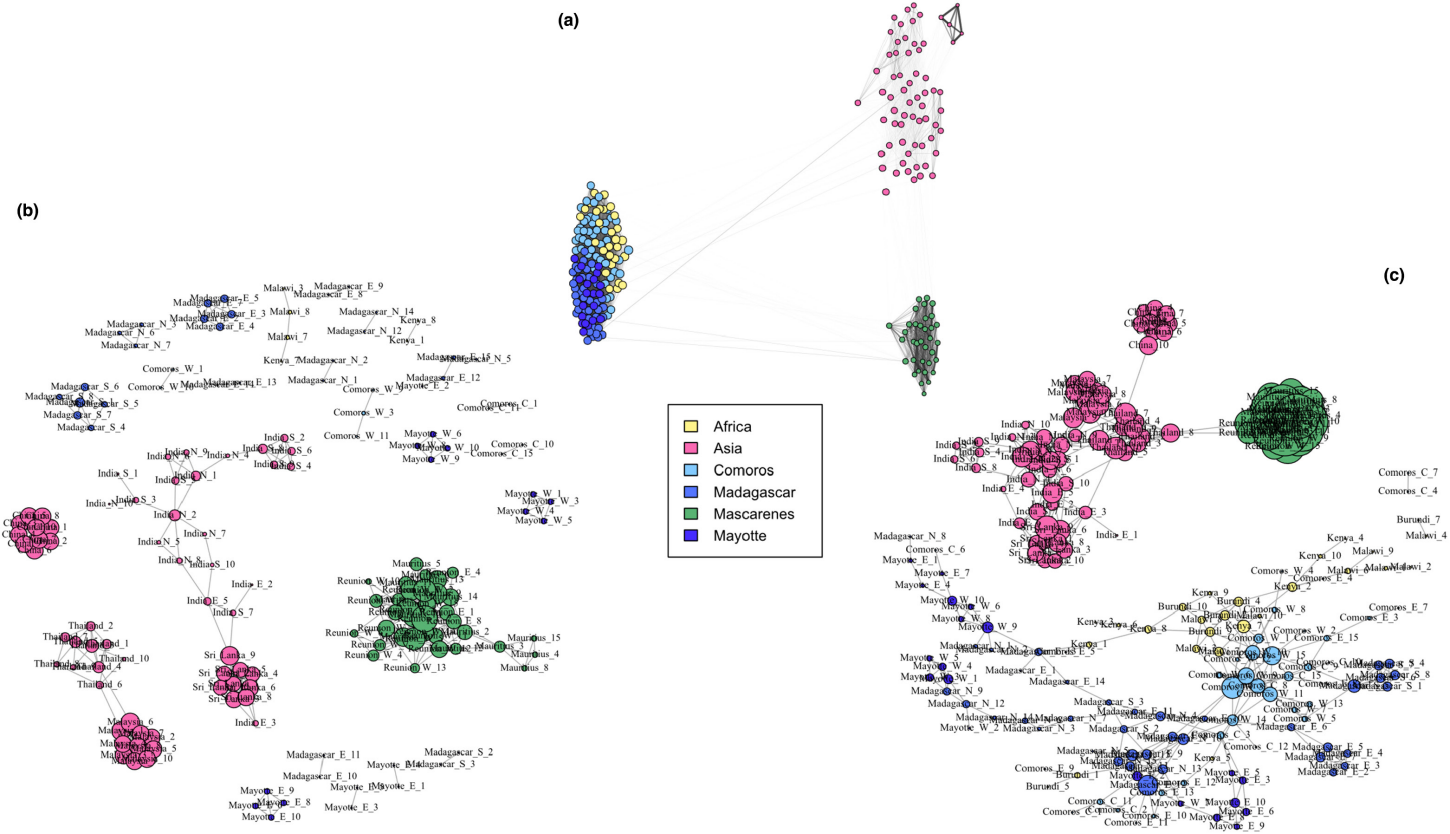
Kinship networks of *Bactrocera dorsalis* in the Indian Ocean in the force‐directed Fruchtermann–Reingold layout. In the first network (a), all edges are represented, and edge opacity and thickness are proportional to the weight of the respective edge. In the second (b) and third network (c), a backbone pruning method was applied, so that only edges with *p‐*value ≤0.05 and 0.10 are conserved, respectively.

Three distinct clusters could be observed when all edges between individuals are conserved (Figure 5a). Samples from the Mascarene islands (Eastern cluster) seem to exhibit a closer proximity to the Asian cluster than samples from Africa, the Comoros, Mayotte, and Madagascar (Western cluster) indicating a higher level of kinship (Figure 5a).

Within the pruned (*p* ≤ 0.05) network, extensive geographic structuring could be observed within the Asian group with cluster outlines largely coinciding with country borders for Sri Lanka, China, Thailand, and Malaysia (Figure 5b). No edges can be observed between the Asian populations and Africa or populations within the Indian Ocean. The Mascarene islands form a distinct and compact cluster with four Mauritian individuals displaying a more peripheral position (Figure 5b). When applying less stringent pruning (*p* ≤ 0.10), a more connected network resulted (600 instead of 387 edges). All Asian populations formed one group sharing two edges with the Mascarenes. Africa, Comoros, Mayotte, and Madagascar constituted another group within the network (Figure 5c). Interestingly, it is the most Western island (Grande Comoro, Table S1) that serves as the primary connector with Africa as exemplified by the large degree of Comoros_W_15 (6) and Comoros_W_3 (5) and other Comoros islands only play a minor role in this. The network structure is less dense within Mayotte and Madagascar and clustering can be observed only to a minor extent.

## 4 DISCUSSION

In light of tracing the origin of invasive fruit fly incursions, researchers have increasingly focused on resolving the population structure of various tephritid species (Deschepper et al., 2021; Dupuis et al., 2018; Kim et al., 2021; Narde et al., 2005; Popa‐Báez et al., 2021; Ruiz‐Arce et al., 2020; Virgilio et al., 2010). For example, by amplifying the *N5N4* mitochondrial region in *Ceratitis capitata*, Ruiz‐Arce et al. (2020) were able to discriminate between six worldwide geographical regions but struggled to provide a more detailed population genetic structure. A comparable approach was used by Kim et al. (2021) and Qin et al. (2018), who used *COI* and *NAD6* haplotype diversity, respectively, in combination with microsatellite markers to infer regional clustering patterns of *B. dorsalis* in a large part of its Asian range. Here, they found evidence for founder effects but did not achieve proof for extensive genetic structure, details that could inform invasion pathways. To our knowledge, our study is the first to use SNP markers resulting from whole‐genome resequencing to examine tephritid population structuring and have high enough resolution to characterize invasion routes. Within the Asian range, we were able to discriminate between populations using a combination of population‐wide allele frequency information in the PoMo analysis and kinship metrics to add granularity at the individual level (Figures 4 and 5). We found populations in Telangana, India (Table S1), and Sri Lanka to be a sister group to populations in Punjab and Tamil Nadu, India (Table S1), China, Thailand, and Malaysia. These results corroborate unpublished work by Y. Zhang, S. Liu, M. De Meyer, M. Virgilio, S. Feng, Y. Qin, S. Singh, S. L. Wee, F. Jiang, S. Guo, X. Zhou, H. Li, P. Deschepper, H. Delatte, A. van Sauers‐Muller, T. S. Syamsudin, A. P. Kawi, M. Kasina, K. Badji, F. Said, L. Liu, Z. Zhao, Z. Li (in preparation) who focus on the worldwide phylogeography of *B. dorsalis*. Since there may be ongoing admixture between the sampled locations, a bifurcating population tree is likely not entirely representative of the individual‐level genetic relationships but rather renders a general perspective on the phylogeny of *B. dorsalis* (De Maio et al., 2015).

The colonization of islands in the Indian Ocean by *B. dorsalis* is a relatively recent phenomenon (Zeng et al., 2019) and is characterized by two independent invasion pathways, as pointed out by distinct partitioning of individuals in all of the analyses performed here (sNMF ancestry estimation, Figure 2; PCA, Figure 3; PoMo tree, Figure 4). The Comoros, Mayotte, and Madagascar, together with the African populations, belonged to one pathway, while the Mascarenes were contained within a separate cluster (Figures 2, 3, 4). Source populations for both colonization pathways are likely different but share genetic variation to some extent. However, fully shared genetically diverse origins cannot be excluded, with drift effects and bottlenecks driving the divergent genetic structure. In the latter case, we would expect to see a larger degree of shared ancestry (Figure 2) in the case of a single origin for both pathways, certainly because the sampling took place only 4–6 years after the invasion of the Mascarenes, rendering little time for drift effects to alter the genetic composition in such a drastic way as observed here. Furthermore, the ML tree reconstruction showed a strong support for two independent invasion routes within the Indian Ocean with a basal position of the Mascarenes (Figure 5).

It has to be noted that every population in this study is characterized by a different time frame between the moment of sampling and the time of colonization, which could bias the amount of genetic differentiation that can be observed between the source and introduced population. However, drift especially acts strongly in the early stages after a population bottleneck and wears off over time (Gilchrist et al., 2012). The fast lifecycle of *B. dorsalis* could imply that drift rapidly changes population genetic structure and differentiation after colonization took place and time and thus only a limited time is needed to pick up signatures of a genetic bottleneck after the invasion.

### 4.1 Western invasion pathway

A western invasion pathway is characterized by step‐wise island hopping starting from the east African coast and ending in Madagascar. Grande Comore (Comoros W) was most likely the first Indian Ocean island to be colonized as suggested by the backbone structure of the kinship network (Figure 5c) and is consistent with the timing of the first observations of *B. dorsalis* on the Comoros (De Meyer et al., 2012). Additionally, the Comorian populations were reconstructed as a paraphyletic group, with Comoros W (Grande Comore, Table S1) as the group most closely related to an African population, namely the Malawi population (Figure 4). International trade data corroborate the link between the Comoros W and the African mainland. The main port of entry and the international airport are situated in Moroni, Grande Comore (Comoros W), where fresh goods are imported from different parts of the world including neighboring countries such as Madagascar, Tanzania, Kenya, or Mozambique (2.6%, 0.8%, 0.6%, and 0.2% of total goods import, respectively) (2020 data from https://OEC.world). Of all Indian Ocean countries, Comoros exhibits the strongest similarity of genetic ancestry with the African region (Figure 2) and has a similar genetic diversity (Table 1), further pointing to Comoros as the western point of entry into the Indian Ocean. From there on, *B. dorsalis* invaded Mayotte and Madagascar resulting in an increase of genetic differentiation to populations of the African mainland (Tables 1 and 2). Additionally, a decrease in the amount of the yellow cluster in the admixture plots is suggestive of genetic drift in the form of founder effects (Figure 2a; Tables 1 and 2). Similarly, Sendell‐Price et al. (2021) found evidence for founder‐induced loss of genetic diversity in an island‐hopping field cricket (*Teleogryllus oceanicus*) and increased genetic differentiation when moving further away from the source. The significantly higher SROH for Mayotte and Madagascar compared with Africa and Comoros further corroborates an eastward direction of invasion coupled with increased inbreeding (Figure 1). Interestingly, the network structure of *B. dorsalis* in Madagascar largely coincides with population delimitation (Figure 5a,c), suggesting that multiple introduction events are at the basis of the Malagasy populations. Alternatively, *B. dorsalis* may have spread through Madagascar while experiencing bottlenecks, which could explain the markedly lower nucleotide diversity in Madagascar S compared with the other Malagasy populations (*π* = 0.0138 vs. 0.0145 and 0.0147).

### 4.2 Eastern invasion pathway

Réunion and Mauritius are always clustered together (Figures 2, 3, 4, 5) and are significantly related (Figure 5b). In contrast to islands along the western invasion pathway, the Mascarene islands were colonized directly from Asia and our results do not suggest a relationship with the western pathway. The weak links between both pathways observed in Figure 5a disappear when backbone pruning is applied and thus suggests the insignificance of these links (Figure 5b,c). Pinpointing an Asian source for *B. dorsalis* on the Mascarene islands is challenging and would require more exhaustive sampling, including all origin countries of fresh fruit imports. For example, trade data reveal that the three largest providers of fresh fruits and vegetables for Mauritius are China, the United Arab Emirates, and India each constituting 17.6%, 11.5%, and 9.9% of the import (2020 data from https://OEC.world). It is known from the literature that *B. dorsalis* has reached Mauritius on several occasions, followed by eradication programs after which the species was considered eradicated from the island (Seewooruthun, Permalloo, et al., 2000; Seewooruthun, Soonnoo, & Alleck, 2000; Sookar & Deguine, 2016). Our data agree with recolonization of the Mascarene islands from a genetically diverse source. If *B. dorsalis* had been in a dormant phase with low numbers, one might expect to see a heavy impact of a bottleneck, which is not what was observed here. While the phylogenetic reconstruction might point toward colonization of Mauritius from Réunion, the more derived position of the Mauritian population in the tree topology might also be explained by the later sampling date of Mauritius compared with Réunion (2021 and 2019, respectively). Furthermore, a surveillance network with methyl eugenol traps has been installed on Réunion island since 2013, and traps were checked every 2 weeks; the first *B. dorsalis* individual was detected in May 2017 on Réunion (Moquet et al., 2021), whereas it was detected in 2015 on Mauritius (Sookar et al., 2020). The ROH analysis revealed that populations on the Mascarene islands are less impacted by a bottleneck than populations on other Indian Ocean islands. However, genetic diversity is lower on the Mascarenes when compared to other island populations. The lower diversity in combination with smaller SROH could indicate that only a limited number of different genotypes were introduced on the Mascarene islands (Figure 1). Hence, the low genetic diversity is a product of the recency of the founder effect rather than long‐term drift on the islands themselves. This is in contrast to Madagascar and Mayotte where the lower genetic diversity compared with the African mainland is likely related to a series of bottlenecks upon introduction on the islands, accompanied by prolonged periods of drift and inbreeding as indicated by the higher SROH (Figure 1). Additionally, the high number of private alleles, both on a regional and at a population level (Table 1), corroborates the lack of drift on the Mascarenes though caution is advised when interpreting the number of private alleles since this statistic is influenced by a priori assignment of samples to groups.

Ultimately, we think that this study could provide policymakers with valuable information to prevent the re‐establishment of *B. dorsalis* after successful eradication or prevent new invasions. Long‐distance dispersal in the case of isolated areas such as oceanic islands is usually linked with human activities. The main driver for the invasion of agricultural pests is still considered to trade (see Hulme, 2021 for a review). Nevertheless, other major pathways such as luggage of airline passengers (Liebhold et al., 2006) and international shipment of goods (both private and through internet commerce, Lenda et al., 2014) are considered of increasing importance. In popular tourist destinations with a large proportion of the local population having foreign roots and family ties, as is the case in the majority of the western Indian Ocean islands, these pathways are even more pertinent. Characterizing invasion pathways can, therefore, assist policymakers in developing appropriate measures and policies to curb further introductions. It will allow them to target particular trade routes and visitors for more scrutinous phytosanitary inspections, which can even be modeled and adapted by taking into account aspects such as traffic peaks, travel, seasons, and temporal abundance of potential pests in countries of origin (Szyniszewska, 2016). Models for a particular horticultural pest such as the one developed in this research can then be extrapolated to other organisms that are similarly associated with the same crops. Moreover, looking at infestation rates and host range of the invasive *B dorsalis* populations from the Mascarenes (Moquet et al., 2021; Sookar et al., 2020) compared with the ones from Madagascar or Comoros (Hassani et al., 2016; Rasolofoarivao et al., 2022) it seems that the Mascarenes populations have slightly different ecological parameters with a broader host range and higher infestation rates, highlighting the importance of preventive measures for pest control.

## 5 CONCLUSION

Our study is the first to investigate the population structure and invasion history of *B. dorsalis* in the Indian Ocean. By using whole genome SNP data, we resolved two independent invasion pathways. A western invasion pathway involves stepping‐stone migration of *B. dorsalis* from the east African coast into Comoros, along Mayotte, and into Madagascar with a decreasing genetic diversity. Our findings are in line with the first detection of *B. dorsalis* on the African mainland in 2003, followed by a relatively quick invasion of Comoros. The Mascarene islands were colonized directly from Asia and formed a distinct cluster. The low nucleotide diversity suggests that only a few genotypes invaded the Mascarenes. The presence of many long runs of homozygosity (ROH) in the introduced populations indicates population bottlenecks, with a more severe bottleneck effect for populations along the western migration pathway than on the Mascarene islands.

## CONFLICT OF INTEREST

All authors declare that they have no conflicts of interest.

## BENEFIT‐SHARING STATEMENT

All geographic samples were collected by the different co‐authors in compliance with their national jurisdiction or sourced from collections obtained prior to the Nagoya Protocol ratification.

## Supporting information


Tables S1–S3

## Data Availability

With the aim of full access and benefit‐sharing within the framework of the Nagoya Protocol, a zenodo data archive was created containing a filtered VCF file, a metadata file, and a count file. The VCF file contains raw SNP information with only the GATK filters applied. The data can be accessed using the following doi: 10.5281/zenodo.6602072. Raw paired‐end read data are stored as a BioProject (PRJNA893460). Admittance to related genetic resources and databases is herewith provided. Research and development results are fully shared by all co‐authors involved, and joint ownership of relevant intellectual property rights is assured.
