# Supplementary material for: Bactrocera dorsalis in the Indian Ocean: A tale of two invasions
Source: Evol Appl. 2022 Dec 1;16(1):48–61. doi: 10.1111/eva.13507 (PMC9850006; doi:10.1111/eva.13507)
Supplement: Supplementary file 1 — Tables S1–S3 [file EVA-16-48-s001.docx]

**Supplemental information**

Table S1: Sample information for every *B. dorsalis and B. zonata* population used in the study.

| **Population** | **group** | **latitude** | **longitude** | **Year** | **country/island** | **Locality** | **#individuals** |
| --- | --- | --- | --- | --- | --- | --- | --- |
| China | Asia | 23.116667 | 113.25 | 2018 | China | Guangdong | 10 |
| India N | Asia | 30.766667 | 75.466667 | 2019 | India | Punjab | 10 |
| India S | Asia | 11 | 78 | 2018 | India | Tamil Nadu | 10 |
| India E | Asia | 17.99 | 79.59 | 2019 | India | Telangana | 6 |
| Malaysia | Asia | 2.888750 | 101.757233 | 2018 | Malaysia | Selangor | 10 |
| Sri Lanka | Asia | 8.333333 | 80.5 | 2019 | Sri Lanka | Anuradhapura | 9 |
| Thailand | Asia | 13.75 | 100.516667 | 2018 | Thailand | Bangkok | 10 |
| Burundi | Africa | -3.36126 | 29.347916 | 2011 | Burundi | Bujumbura | 9 |
| Kenya | Africa | -1.286389 | 36.817223 | 2018 | Kenya | Nairobi | 10 |
| Malawi | Africa | -15.383333 | 35.333333 | 2011 | Malawi | Zomba | 10 |
| Comoros E | Comoros | -12.169581 | 44.400469 | 2020 | Anjouan | Mutsamudu | 15 |
| Comoros W | Comoros | -11.887344 | 43.406058 | 2020 | Grande Comore | Mandzissani | 15 |
| Comoros C | Comoros | -12.291428 | 43.742986 | 2020 | Mohéli | Fomboni | 15 |
| Mayotte E | Mayotte | -12.82773 | 45.2 | 2019 | Mayotte | Dembéni | 10 |
| Mayotte W | Mayotte | -12.861036 | 45 | 2019 | Mayotte | Sada | 10 |
| Madagascar E | Madagascar | -18.87855 | 47.55586667 | 2017 | Madagascar | Ambatobe | 15 |
| Madagascar S | Madagascar | -22.89465 | 44.34668333 | 2017 | Madagascar | Mahaboboka | 8 |
| Madagascar N | Madagascar | -15.6440833 | 46.40065 | 2016 | Madagascar | Mangatsa | 15 |
| Réunion W | Mascarenes | -21.322553 | 55.485116 | 2019 | Réunion | Saint-Pierre | 15 |
| Réunion E | Mascarenes | -21.129301 | 55.797422 | 2019 | Réunion | Sainte-Rose | 5 |
| Mauritius | Mascarenes | -20.19031 | 57.72659 | 2021 | Mauritius | La Source | 15 |

Table S2: Pairwise *F_ST_* for *B. dorsalis* populations.

| **Region** | **Burundi** | **China** | **Comoros E** | **Comoros W** | **Comoros C** | **India N** | **India S** | **India E** | **Kenya** | **Madagascar E** | **Madagascar S** | **Madagascar N** | **Malawi** | **Malaysia** | **Mauritius** | **Mayotte E** | **Mayotte W** | **Réunion W** | **Réunion E** | **Sri Lanka** | **Thailand** |
| --- | --- | --- | --- | --- | --- | --- | --- | --- | --- | --- | --- | --- | --- | --- | --- | --- | --- | --- | --- | --- | --- |
| **Burundi** |  | 0.044 | 0.030 | 0.033 | 0.032 | 0.042 | 0.051 | 0.059 | 0.034 | 0.042 | 0.058 | 0.040 | 0.037 | 0.044 | 0.058 | 0.050 | 0.050 | 0.055 | 0.075 | 0.052 | 0.042 |
| **China** |  |  | 0.042 | 0.043 | 0.043 | 0.030 | 0.034 | 0.038 | 0.044 | 0.045 | 0.050 | 0.045 | 0.045 | 0.030 | 0.036 | 0.048 | 0.049 | 0.035 | 0.039 | 0.034 | 0.030 |
| **Comoros E** |  |  |  | 0.026 | 0.021 | 0.040 | 0.047 | 0.054 | 0.028 | 0.029 | 0.038 | 0.028 | 0.031 | 0.042 | 0.051 | 0.032 | 0.034 | 0.049 | 0.060 | 0.048 | 0.040 |
| **Comoros W** |  |  |  |  | 0.025 | 0.041 | 0.048 | 0.056 | 0.032 | 0.036 | 0.047 | 0.034 | 0.030 | 0.043 | 0.053 | 0.040 | 0.040 | 0.051 | 0.063 | 0.049 | 0.041 |
| **Comoros C** |  |  |  |  |  | 0.041 | 0.048 | 0.056 | 0.030 | 0.029 | 0.040 | 0.028 | 0.031 | 0.043 | 0.053 | 0.033 | 0.036 | 0.050 | 0.063 | 0.049 | 0.041 |
| **India N** |  |  |  |  |  |  | 0.032 | 0.036 | 0.042 | 0.043 | 0.048 | 0.043 | 0.043 | 0.030 | 0.035 | 0.046 | 0.046 | 0.034 | 0.038 | 0.033 | 0.029 |
| **India S** |  |  |  |  |  |  |  | 0.042 | 0.051 | 0.052 | 0.059 | 0.051 | 0.052 | 0.034 | 0.041 | 0.056 | 0.057 | 0.039 | 0.046 | 0.038 | 0.032 |
| **India E** |  |  |  |  |  |  |  |  | 0.058 | 0.060 | 0.069 | 0.058 | 0.060 | 0.038 | 0.049 | 0.065 | 0.066 | 0.047 | 0.057 | 0.043 | 0.036 |
| **Kenya** |  |  |  |  |  |  |  |  |  | 0.039 | 0.052 | 0.037 | 0.034 | 0.044 | 0.057 | 0.044 | 0.046 | 0.054 | 0.070 | 0.052 | 0.042 |
| **Madagascar E** |  |  |  |  |  |  |  |  |  |  | 0.040 | 0.024 | 0.040 | 0.045 | 0.057 | 0.033 | 0.036 | 0.055 | 0.070 | 0.053 | 0.043 |
| **Madagascar S** |  |  |  |  |  |  |  |  |  |  |  | 0.036 | 0.055 | 0.050 | 0.070 | 0.051 | 0.055 | 0.065 | 0.094 | 0.060 | 0.048 |
| **Madagascar N** |  |  |  |  |  |  |  |  |  |  |  |  | 0.037 | 0.045 | 0.056 | 0.031 | 0.032 | 0.053 | 0.067 | 0.052 | 0.043 |
| **Malawi** |  |  |  |  |  |  |  |  |  |  |  |  |  | 0.045 | 0.059 | 0.046 | 0.047 | 0.056 | 0.074 | 0.053 | 0.043 |
| **Malaysia** |  |  |  |  |  |  |  |  |  |  |  |  |  |  | 0.036 | 0.048 | 0.049 | 0.035 | 0.039 | 0.034 | 0.029 |
| **Mauritius** |  |  |  |  |  |  |  |  |  |  |  |  |  |  |  | 0.065 | 0.066 | 0.025 | 0.035 | 0.042 | 0.035 |
| **Mayotte E** |  |  |  |  |  |  |  |  |  |  |  |  |  |  |  |  | 0.043 | 0.061 | 0.083 | 0.057 | 0.046 |
| **Mayotte W** |  |  |  |  |  |  |  |  |  |  |  |  |  |  |  |  |  | 0.062 | 0.085 | 0.058 | 0.046 |
| **Réunion W** |  |  |  |  |  |  |  |  |  |  |  |  |  |  |  |  |  |  | 0.033 | 0.040 | 0.034 |
| **Réunion E** |  |  |  |  |  |  |  |  |  |  |  |  |  |  |  |  |  |  |  | 0.048 | 0.037 |
| **Sri Lanka** |  |  |  |  |  |  |  |  |  |  |  |  |  |  |  |  |  |  |  |  | 0.033 |

Table S3: Post-hoc comparisons of the parametric linear model comparing SROH between regions.

| **Post-hoc comparison** | **Estimate** | **Std. Error** | **z value** | **Pr(>\|z\|)** | **sign.** |
| --- | --- | --- | --- | --- | --- |
| Asia - Africa | -59726955 | 2707272 | -22.062 | <0.001 | *** |
| Comoros - Africa | 7005086 | 2886917 | 2.426 | 0.144 |  |
| Madagascar - Africa | 20179229 | 2989305 | 6.75 | <0.001 | *** |
| Mascarenes - Africa | -21759824 | 3044252 | -7.148 | <0.001 | *** |
| Mayotte - Africa | 15901873 | 3523771 | 4.513 | <0.001 | *** |
| Comoros - Asia | 66732041 | 2351024 | 28.384 | <0.001 | *** |
| Madagascar - Asia | 79906185 | 2475676 | 32.277 | <0.001 | *** |
| Mascarenes - Asia | 37967132 | 2541751 | 14.937 | <0.001 | *** |
| Mayotte - Asia | 75628829 | 3099998 | 24.396 | <0.001 | *** |
| Madagascar - Comoros | 13174143 | 2670943 | 4.932 | <0.001 | *** |
| Mascarenes - Comoros | -28764910 | 2732300 | -10.528 | <0.001 | *** |
| Mayotte - Comoros | 8896787 | 3258060 | 2.731 | 0.0674 |  |
| Mascarenes - Madagascar | -41939053 | 2840268 | -14.766 | <0.001 | *** |
| Mayotte - Madagascar | -4277356 | 3349121 | -1.277 | 0.7938 |  |
| Mayotte - Mascarenes | 37661697 | 3398255 | 11.083 | <0.001 | *** |
